# Enhancing the sensitivity of nanopipette biosensors for protein analysis

**DOI:** 10.1002/brb3.3405

**Published:** 2024-02-04

**Authors:** Mustafa Demirtas

**Affiliations:** ^1^ Department of Electrical and Electronics Engineering Bursa Uludağ University Bursa Türkiye

**Keywords:** nanopipette, nanopore, biosensor, protein translocation, numerical simulations, single‐protein analysis, proteomics

## Abstract

**Background:**

This paper compares experimental findings and simulation outcomes of single and multiple protein models moving through a nanopipette biosensor. It provides insights into the factors influencing the process and explores their relevance to proteomics.

**Methods:**

Nanopipette biosensors were produced by pulling borosilicate glass tubes and treating them with an electron beam. A scanning electron microscope was used to characterize the nanopipettes. The study measured and modeled ionic currents for the elastase‐specific inhibitor protein. Simulation models were developed using the finite element method and Poisson–Boltzmann formalism, considering different protein configurations and translocation scenarios.

**Results:**

The results showed that the pore current of a nanopipette decreases as the protein approaches the nanopipette. The minimum pore current occurs at the widest part of the protein, and the current increases as the protein progresses through the nanopipette. For multiple protein translocations, the pore current decreases between the widest parts of the first and second proteins, and the lowest current is observed at the broadest part of the second protein. After the third protein, the pore current remains constant. It is also found that the fractional blockade difference, translocation speed, fluctuation in pore current, and dwell time are all affected by the number of proteins translocating through the nanopipette. The fractional blockade difference, the decrease in pore current caused by the protein, increases with the number of proteins while the translocation speed decreases. The fluctuation in pore current and dwell time is also longer for three‐protein translocations than for single‐protein translocations.

**Conclusion:**

This study offers valuable insights into biomolecule transport through nanopipettes, enhances our understanding of protein dynamics in restricted environments, and significantly contributes to single‐protein sequencing studies, drug screening, and proteomics.

## Graphical abstract

This paper compares experimental and simulation results on the movement of single and multiple proteins through a nanopipette biosensor. The study aims to understand the factors influencing protein translocation and its implications for proteomics. The findings indicate that finite element method simulations accurately predict the transport of proteins.

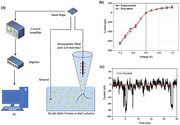

## 1 INTRODUCTION

Nanopipette biosensors are electrochemical devices at the nanoscale that have been gaining increasing popularity in various applications, such as nanoscale biosensing, nanoparticle detection, and conducting reactions in confinement (Edwards et al., 2015; Fu & Bohn, 2018; Lin et al., 2018; Perry, Parker, et al., 2016; Zhang et al., 2018). To effectively control reaction conditions within a nanopipette, it is crucial to understand the local electric field and ion distribution. The current understanding in this area is primarily based on simulations using the Poisson–Nernst–Planck model (PNP) (Cressiot et al., 2012; Innes et al., 2010; Morris, McPherson, Edwards, et al., 2020; Morris, McPherson, Meloni, et al., 2020; Perry, Parker, et al., 2016; Qiao et al., 2014). The Navier–Stokes equation and speciation reactions are added to account for electroosmotic flow and solution equilibria (Sa & Baker, 2013; White & Bund, 2008). However, these models often make assumptions that have not been thoroughly tested, such as assuming infinitely thick pore walls (Sa & Baker, 2013). Thin nanopipette walls, for example, can generate significant capacitance and affect the potential at the wall, leading to charge accumulation and electroosmosis of the second kind (McPherson et al., 2021).

The transport of biomolecules such as proteins through nanopipettes has attracted significant interest due to its potential applications in biosensing and drug delivery (Clarke et al., 2005; Karhanek et al., 2005; Ren et al., 2017; Umehara et al., 2009). However, controlling the translocation process, particularly in the case of multiple proteins, remains a significant challenge. Although some studies have investigated the transport of individual proteins through nanopipettes, there is a lack of understanding of how multiple proteins affect each other's transport rates and overall transport behavior.

Coulter counters are instruments employed to characterize and count particles, particularly in the micron size range (0.1−1000 μm). They operate on the Coulter principle, which involves measuring changes in ionic current flow through micron‐sized channels as particles pass through. When particles obstruct this flow, they cause a transient reduction in the ionic current, known as a current blockade, with its magnitude and duration directly linked to the particle's size. Larger particles create more significant and longer‐lasting blockades, whereas smaller particles cause minor and shorter‐lived disruptions in the current. By measuring these changes in current, Coulter counters can accurately determine the size and count of particles within a sample (Kozak et al., 2011).

The translocation of a protein through a nanopipette depends on various factors, such as the protein's size, shape, charge, and environmental conditions. Some of these factors can slow the translocation process by increasing the interaction between the protein and the nanopore or making the protein more challenging to pass through the nanopore. For example, larger, complex, positively charged, unfolded proteins will take longer to translocate than smaller, simple, negatively charged, folded proteins. Similarly, higher temperature, pH, and ionic strength will increase the translocation time by affecting the protein's motion, structure, and viscosity. This information is critical for developing single‐protein sequencing, drug screening, and delivery studies.

Nanopipette biosensors have also been used as promising tools for drug sensing and delivery applications, mainly due to their ability to probe single cells with high precision and minimal invasiveness. Functionalizing nanopipettes with specific recognition elements enables the creation of highly selective drug sensors, allowing for detecting minute concentrations of drugs in complex biological matrices. These sensors offer the potential for real‐time monitoring of drug levels in patients' bodies, facilitating personalized medicine and optimized dosing while minimizing side effects. In recent research by Ruan et al. (2021), an organic molecule/NiO/Ni film was engineered at the tip of a nanopipette to realize a dual‐functional photoelectrochemistry (PEC) single‐cell nanotool toward direct intracellular delivery and evaluation of oxidative stress. Additionally, Actis et al. (2014) demonstrated the electrochemical capabilities of nanopipettes for single‐cell analysis, highlighting their relevance in the drug sense. However, ongoing challenges in improving sensor stability, reproducibility, and scalability need to be addressed to harness the potential of nanopipette biosensors entirely in drug sensing.

Introducing low concentrations of similar molecules, such as polyether compounds, can potentially impact the translocation dynamics of biomolecules through disruption of electroosmotic outflow. For example, one study found that adding a low concentration of a polyether compound to the electrolyte solution could increase the translocation speed of DNA molecules through a nanopore (Ermann et al., 2018). Another study found that the addition of a low concentration of a polyether compound to the electrolyte solution could alter the translocation dynamics of DNA molecules through a nanopore in a way that is dependent on the length of the DNA molecule (Chau et al., 2020). One another research investigates ionic conductance through nanopores, uncovering deviations from bulk behavior at low salt concentrations due to salt‐dependent surface charges. Furthermore, the study demonstrates that DNA translocation can be employed to estimate DNA volume and effective charge, expanding the utility of nanopores in characterizing various molecules and interactions (Smeets et al., 2006).

We used a modified glass nanopipette with a small orifice to confine protein molecules and monitored their translocation through the nanopore using electrical measurements to achieve our objectives. We also performed finite element method (FEM) simulations to predict the transport of single and multiple proteins through the nanopipette and compared the simulation results with our experimental measurements. We found that the transport of multiple proteins can be accurately predicted using FEM simulations, with good agreement between the simulated and experimental pore currents. Our results also show that the presence of multiple proteins can affect the transport of individual proteins, with some proteins exhibiting a reduced transport rate in the presence of other proteins.

We compare our approach with existing research efforts on nanopipette biosensors and electrokinetic phenomena in nanopores. Table 1 summarizes the main features and findings of different theoretical models and experimental studies in the literature. We highlight the advantages and limitations of each method, as well as the gaps that our work aims to fill. Our results demonstrate that our nanopipette biosensor can achieve high sensitivity and selectivity for protein detection.

**TABLE 1:** Comparison of previous literature studies and this study.

Reference	NP material	NP geometry	Electrokinetic phenomena	Theoretical model	Experimental method	Sensitivity enhancement
Zhang et al. (2017)	Glass	Cylindrical	Ionic current blockade	Nernst–Planck equation	Ionic current measurement	Dependent on DNA length, pore size, and ionic strength
Rabinowitz et al. (2019)	Quartz	Conical	Ion current rectification	Navier–Stokes equation	Ionic current and impedance measurement	Caused by nonlinear electroosmotic flow and nanoscale fluid vortices
Lan et al. (2016)	Glass	Conical	Voltage‐rectified current and fluid flow	Poisson–Nernst–Planck–Stokes equation	Ionic current and electroosmotic pumping measurement	Related to ion concentration polarization, energy conversion, and biosensing
Perera et al. (2015)	Glass	Conical	The activation energy of ion transport	Poisson–Boltzmann equation	Ionic current and temperature measurement	Influenced by electric potential and ion concentration
Luo et al. (2012)	Glass	Conical	Negative differential electrolyte resistance	Poisson–Nernst–Planck equation	Ionic current measurement	Resulted from the interplay of electric field, ion concentration, and fluid flow
White and Bund (2008)	Glass	Conical	Ion current rectification	Poisson–Boltzmann equation	Ionic current measurement	Dependent on pore geometry, surface charge, and Debye length
Wei et al. (1997)	Quartz	Conical	Ion current rectification	Poisson–Boltzmann equation	Ionic current and impedance measurement	Caused by asymmetric distribution of surface charge and electric double layer
This study	Glass	Conical	Protein detection	Poisson–Boltzmann Nernst–Planck equation	Ionic current measurement	Enhanced by nanopipette functionalization and signal processing

This study provides valuable insights into the complex interplay between multiple proteins during transport through nanopipettes and advances our understanding of protein transport in confined spaces. These findings have important implications for developing nanopipette‐based biosensors and drug delivery systems and will stimulate further research.

## 2 MATERIALS AND METHODS

The nanopipette biosensors were produced by pulling borosilicate glass tubes (inner diameter: 0.3 mm, outer diameter: 1.0 mm, length: 10 cm, catalog number: B100‐30‐7.5HP, Sutter instrument) using a laser puller (P‐2000, Sutter Instruments). Before pulling, organic residues were removed from the glass tubes using a (blue–white) 25 W oxygen plasma (4:1) O_2_:N_2_ at a chamber pressure of 1500 mTorr for 4 min (Harrick Plasma).

The laser puller focuses a high‐energy CO_2_ laser beam onto the center of the glass tube, which reaches the melting point of glass and applies a mechanical pulling force simultaneously. This highly reproducible process results in the production of two identical nanopipettes with a sharp tip. The recipe used to produce nanopipettes is as follows:

(1) HEAT: 735; FIL: 4; VEL: 30; DEL: 170; PUL: 80,

(2) HEAT:800; FIL:3; VEL:20; DEL:175; PUL:180.

The shrinking nanopore diameter at the nanopipette tip under electron‐beam exposure is crucial for achieving high sensitivity and selectivity in resistive pulse sensing of proteins. The nanopore diameter determines the magnitude and shape of the current blockades induced by the translocation of proteins through the nanopore. Therefore, it is essential to have precise control over the nanopore diameter and to monitor it during the shrinking process. The e‐beam shrinking method allows us to adjust the nanopore diameter to the desired value by varying the exposure time and the beam parameters (Steinbock et al., 2013). Moreover, the e‐beam shrinking method preserves the conical shape of the nanopipette tip, which is essential for maintaining a high signal‐to‐noise ratio (SNR) and avoiding clogging of the nanopore. After pulling, the nanopipette's cone angles of the waist and orifice are 2° and 13°, respectively. The ion currents in voltage clamp configuration were recorded using Axopatch 200B patch‐clamp amplifier, digitized using Digidata 1440A (Molecular Devices, LLC), and acquired using pClamp 10.7 software (Molecular Devices, LLC). All experiments were performed at room temperature.

Elastase‐specific inhibitor (Gene: PI3, *Homo sapiens*, 117 amino acids, 50 pM concentration) was analyzed by measuring the blockade currents through the nanopipette biosensor.

**FIGURE 1:**
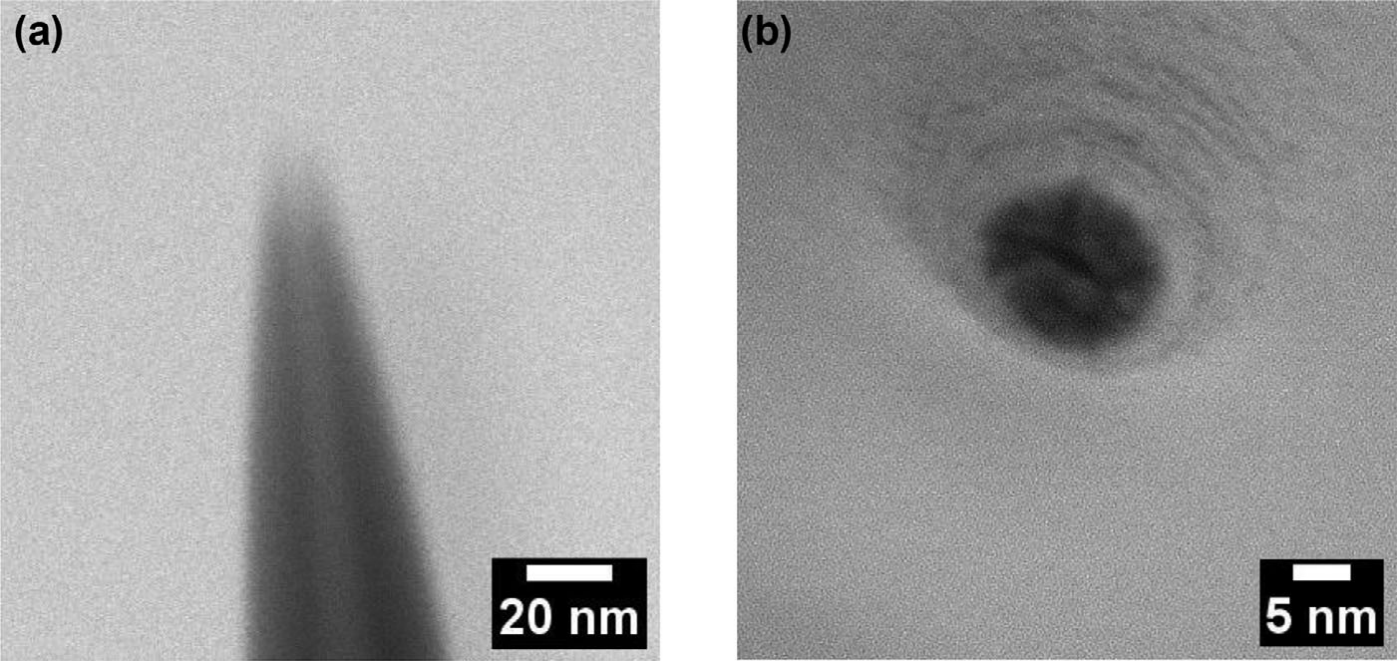
Characterization of nanopipette produced using glass capillary. (a) Scanning electron microscope (SEM) image of the glass nanopipette showing a nanopore with a diameter of 4.6 ± 0.2 nm. (b) The bottom–up view of the same nanopipette is given in (a).

Before being filled with conductive solution, the nanopipettes were characterized using a scanning electron microscope (SEM). This provided information about the tip openings of the nanopipettes to be used as biosensors. The SEM parameters were selected as 4.00 kV and 7.8 pA for high voltage and beam current, respectively, under which the shape and size of the nanopore at the tip of the nanopipette were assumed to remain unchanged. Measurements performed on different nanopipettes produced under similar conditions and with the same measurement parameters showed that the dimension of the gap at the tip of the biosensor was 4.6 ± 0.2 nm. SEM images of the nanopipettes are provided in Figure 1a,b.

In order to measure the open pore current of the biosensor, I–V measurements were performed. A sequence of voltages (V) is applied to the device for an I–V curve measurement, and the open pore current (I) is measured. For the pore current to flow, the inside and outside solutions of the nanopipette must come into contact with each other. To dissolve the proteins and fill the pipette inside, 125 mM NaCl was used. The conductive solution must reach the tip of the pipette without any air bubbles in the nanopipette. This process becomes challenging with the narrow tip of the nanopipette. This process can be accomplished with a micro‐filling needle for micron‐sized tips, but as the tips used in this study were on the order of nm, extra steps were necessary. For this process to be flawless, after the pulling process, the nanopipettes were placed in a petri dish and filled with 125 mM NaCl solution up to the top. Usually, they are left in a desiccator overnight to ensure that the nanopipette is thoroughly wetted up to the tip (Sun et al., 2019).

To ensure the reliability and reproducibility of our experimental setup, we took meticulous precautions to prevent the formation of air bubbles within the nanopipette tip. In detail, we utilized high‐purity solvents and subjected them to thorough degassing processes before nanopipette filling. Care was taken during the filling process to minimize the introduction of air, and we maintained a controlled environment to reduce the risk of air entrapment further. Moreover, the inspection and verification of the nanopipette's narrow tip were carried out with utmost precision. We employed a microscope with high‐resolution imaging to visually confirm the tip's integrity and dimensions. Additionally, we followed a stringent set of criteria to ensure that the nanopipette tip met the required quality standards for our experiments. To address this concern, a series of test measurements were conducted using a 125 mM NaCl solution without proteins. We applied three essential criteria to validate the nanopipette's integrity. First, we rigorously ensured that the open pore current remained stable and free from fluctuations. Second, it was vital that the amplitude of the current measured for each nanopipette consistently fell within the range of ±1 V, thus confirming uniform measurements across various nanopipettes under identical experimental conditions. Lastly, the third criterion dictated that peak‐to‐peak noise did not exceed 30 pA. These stringent measures were rigorously adhered to, minimizing the potential for air bubble formation and fortifying our experimental results' reliability and precision.

**FIGURE 2:**
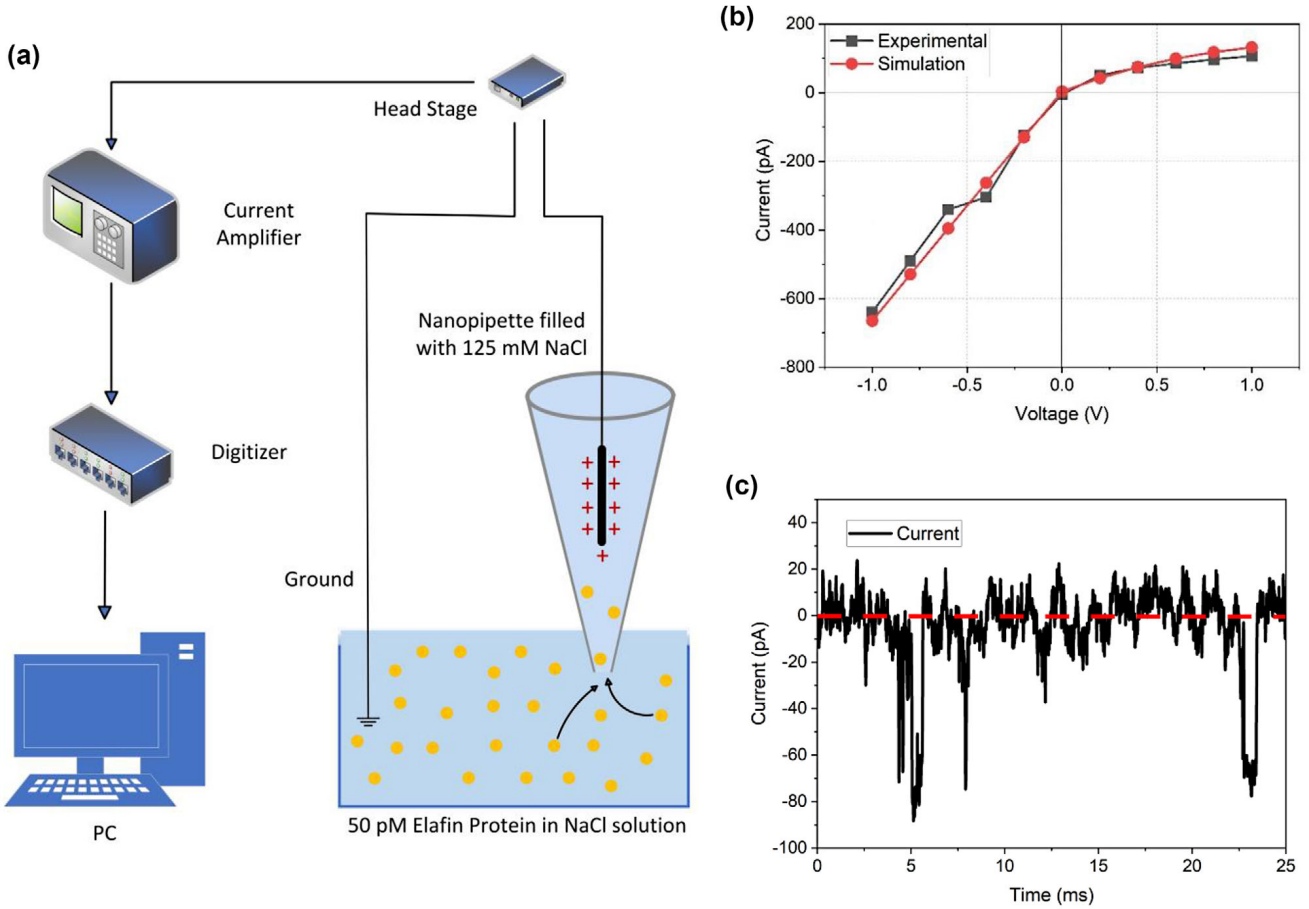
Schematic of setup, representative data for IV, and protein translocation through a nanopipette. (a) Nanopipette (4.6 nm pore diameter), filled with 125 mM NaCl, is immersed in a solution of the same electrolyte with/without the presence of protein. Applying a positive potential to an Ag/AgCl quasi‐reference electrode inside the nanopipette concerning a ground electrode in the external solution causes the migration of proteins. (b) Experimental (black) and simulated (red) IV curves of the nanopipette without proteins in 125 mM NaCl. (c) A representative pore current trace from proteins elastase‐specific inhibitor (ELAFIN) in 125 mM NaCl shows the definition of the blockade and open pore current (red).

The characteristics of pore current were measured using a specially designed Faraday cage setup with a current amplifier and digitizer based on a reverse optical microscope. Figure 2a shows the setup used to measure protein translocation. The pore current was generated using 125 mM NaCl to provide ion flow between the nanopipette's interior and exterior. The experimental setup featured a two‐electrode configuration using a 0.002‐in. (0.051 mm) diameter with 99.99% pure Ag wire inside the nanopipette as the working electrode and a commercial Ag/AgCl reference electrode as the ground. The Ag wire was prepared by immersing it in a sodium hypochlorite solution (NaClO, 95%) for 2 min, ensuring uniform AgCl layer formation. Before each experiment, the Ag/AgCl reference electrode was conditioned in the same solution for 10 min. This pre‐cleaning step was essential for stabilizing and conditioning the Ag wire, promoting the uniform formation of the AgCl layer crucial for accurate electrochemical measurements. A versatile head stage with active cooling provided stable voltage recordings. Standardized 35 mm plastic petri dishes maintained consistent electrode distances.

The signal from this electrode was sent to the head stage, which was used to reduce noise. The head stage created the voltage difference, and the output was connected to a ground connection coated with Ag/AgCl and submerged in the external solution containing 125 mM NaCl. The analog current signal obtained from the head stage was first subjected to analog amplification and then digitized. The analog sampling rate was 100 kHz, and the digitizer sampling rate was 250 kHz. The Nyquist theorem is used for appropriate selections. The digitized current information was collected and stored by the computer for analysis. The I–V response obtained from the glass nanopipette when there was no protein in the solution is shown in Figure 2b. The IV measurements were made in the range of ±1 V to validate the experimental findings and simulation results. The nonlinear IV response had a pore current rectification rate of *R* = 2.57 (± 0.2 V). This is consistent with other studies in the literature on negatively charged glass nanopores (Xiong et al., 2019). This asymmetry is traditionally due to a combination of two effects: (i) the asymmetrical geometry of the end, which causes a difference in the constraining transport rates inside and outside the nanopore, and (ii) surface charge, which leads to permeability for chloride ions (Perry, Momotenko, et al., 2016; Wei et al., 1997).

The measured pore current showing protein translocation after adding the protein to the solution is given in Figure 2c. After the data collection, a 2 kHz eight‐pole Bessel low pass filter was applied to increase the SNR. Background correction was performed, and the open pore current was normalized to have an average of zero. During protein passage through the nanopipette, it blocks the existing ion current, causing it to drop, and this situation continues during the translocation period.

The filter's objective is to maximize the flatness of the group delay curve at a specific frequency. This is important because the group delay is the time it takes for a signal to travel through a filter. If the group delay is not flat, then different signal frequency components will travel through the filter at different speeds, which can distort the signal. There are several ways to design a filter to maximize the flatness of the group delay curve. One common approach is to use a Bessel filter, which we used. Bessel filters are designed to have a maximally flat group delay curve in the passband. Moreover, Bessel filters have a relatively high roll‐off rate, which can significantly attenuate high‐frequency noise. The filter can have a significant impact on the SNR of the signal. The SNR is the ratio of the desired signal to the noise. A higher SNR means the signal is more easily distinguished from the noise. The filter can affect the SNR in two ways. First, the filter can attenuate the noise signal. This can improve the SNR by reducing the noise power. Second, the filter can amplify the desired signal and background correction. This can also improve the SNR by increasing the signal power. Once the background signal has been corrected, the open pore current can be normalized to have an average of zero. This is done by dividing the open pore current by its average value. This ensures that the open pore current has a mean value of zero, regardless of the amplifier's offset or the recording electrodes. Normalizing the open pore current to have an average of zero is essential for several reasons. First, it allows for easier comparison of data from different experiments. Second, it can help to improve the accuracy of measurements. For example, if the open pore current is not normalized, the amplitude of a current pulse will be affected by the amplifier's offset.

The amount and duration of the drop in the pore current vary depending on the properties of the protein used (Hu et al., 2020; Mitscha‐Baude et al., 2021). However, it was observed that even in uniform protein translocation, there were different translocation times despite similar current drop rates. Due to the large population of proteins in the solution, some proteins can cling to each other and translocate together through the biosensor. Whether these translocations are from single or multiple proteins can be distinguished by modeling. The translocation models of the single and multiple proteins connected with a chain model were simulated.

**FIGURE 3:**
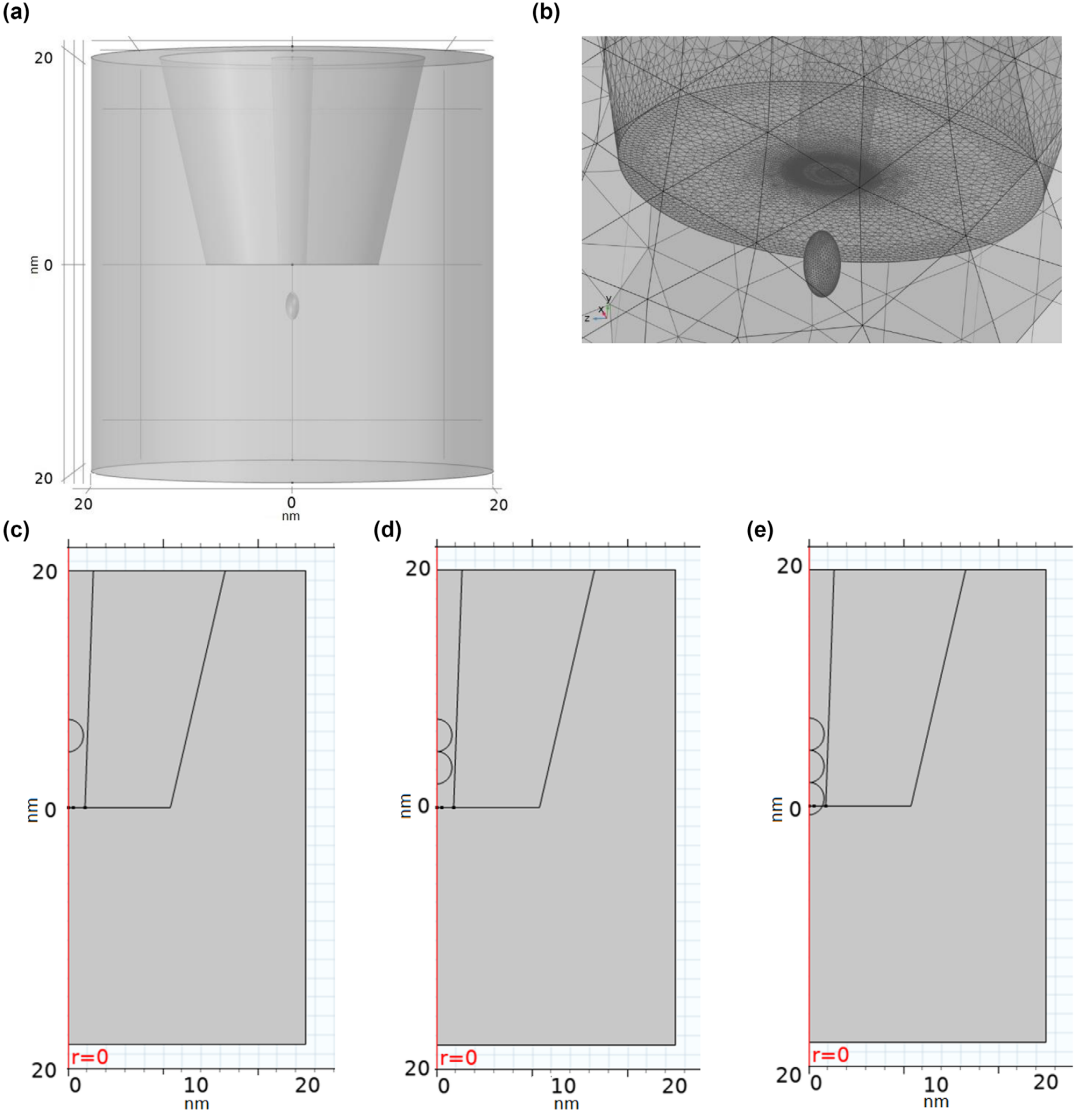
Modeling of protein translocation through nanopipette biosensor. The modeled protein is elastase‐specific inhibitor (ELAFIN). The concentration inside and outside of the nanopipette solution is 125 mM NaCl. The protein is modeled as an ellipsoid shape with 3.141 × 3.403, 1.694 nm dimensions. The surface charge density and protein concentration are 0.012 C/m^2^ and 50 pM, respectively: (a) nanopipette tip and single protein with prolate; (b) nanopipette pore and single protein finite element method (FEM) mesh distribution (extra dense); (c) single‐protein model; (d) 2‐protein model; (e) 3‐protein model. (c–e) Shows a cross‐sectional view of the model for prolate protein.

The electric field's FEM and the electroosmotic flow were performed following a Poisson–Boltzmann formalism described elsewhere (Tang et al., 2006). Single, 2‐, and 3‐protein translocations were modeled. The proteins' surface charge density (SCD) was calculated using the surface area information and protein sequences obtained from “UniProt.” Each protein is modeled separately and has the same characteristics as the other. Simulations were made for both oblate and prolate protein configurations. Multiple protein models assume that all proteins touch each other, line up like a chain, and are made based on moving together. The proteins and translocations are modeled in 3D. The model was verified by changing the input parameters and comparing the open pore current experimental data between ±1 V in the step of 0.2 V. The nanopipette is modeled using SEM images. A 3D model of a nanopipette biosensor and single protein is given in Figure 3. The whole simulation window is shown in Figure 3a. The element sizes of the protein and nanopipette pore are extra fine mesh, shown in Figure 3b. The simulation window is ±20 nm in length in vertical and horizontal planes. The tip of the nanopipette biosensor is located at the center of the simulation window (0 nm). Protein translocation was simulated from 10 nm away from the pore entrance to 15 nm inside the pore in steps of 0.01 nm, and pore current values were calculated during the simulation. The following values were used to run the simulation (Table 2):

**TABLE 2:** Parameters used for simulation.

Diffusivity Na^+^ (m^2^/s)	1.33E‐09
Diffusivity Cl^−^ (m^2^/s)	2.03E‐09
The density of protein (gr/cm^3^)	1.35
The SCD of glass (C/m^2^)	−0.032
The SCD of protein (C/m^2^)	0.012 and 0.065
Glass thickness at orifice (nm)	9
Pore diameter (nm)	4.6
Cone angle waist (°)	2
Cone angle orifice (°)	13
NaCl molarity (mM)	125
Glass relative permittivity	3.9
Water relative permittivity	78.5
Protein relative permittivity	60
Ion radius (nm)	0.15
Voltage (V)	1
Protein dimensions (a‐semiaxis × b‐semiaxis)	1.7015 nm × 1.5705 nm

Abbreviation: SCD, surface charge density.

In choosing our modeling framework, it is essential to provide a comprehensive justification for utilizing the PNP model and the Navier–Stokes equation. The PNP model was selected for its ability to describe the behavior of ions in electrolyte solutions, particularly in scenarios where electrostatic and concentration effects are prominent. The Poisson equation quantifies the electrostatic potential, a fundamental component in understanding charge distribution and interactions within our system. Meanwhile, the Nernst–Plank equations are vital for capturing ion motion, accounting for drift due to electric fields and diffusion due to concentration gradients. This dual approach allows for a detailed description of ion transport and distribution, which aligns perfectly with the primary focus of our study. Similarly, the choice of the Navier–Stokes equation is indispensable for characterizing the motion of incompressible fluids within our system. This equation captures the conservation of momentum in fluid systems and provides essential insights into how ions influence fluid motion and vice versa. The synergistic use of the PNP model and the Navier–Stokes equation forms the backbone of our study, enabling a comprehensive investigation into the coupled dynamics of ions and incompressible fluids.

## 3 RESULTS AND DISCUSSION

**FIGURE 4:**
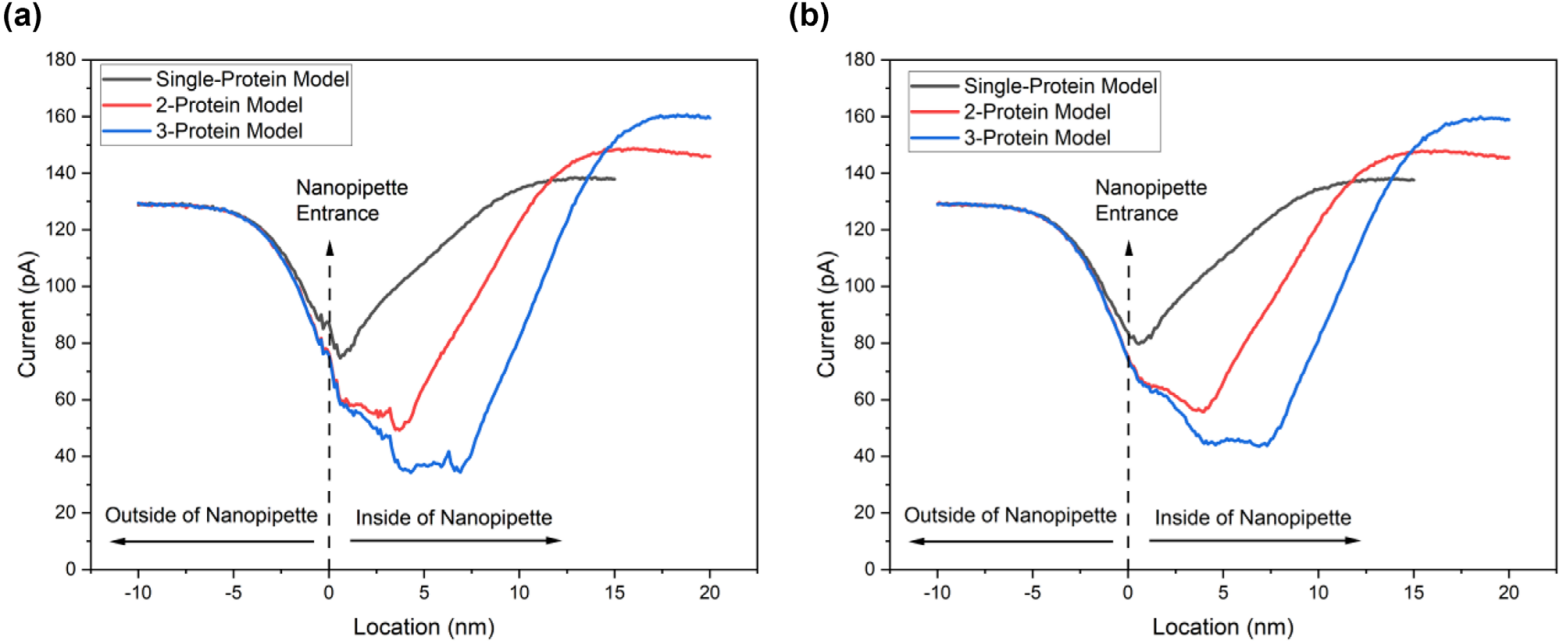
Simulation results of single, 2,‐ and 3‐protein models for both oblate and prolate translocation: (a) oblate single, 2‐, and 3‐protein model results; (b) prolate single, 2‐, and 3‐ protein model results. The surface charge density (SCD) of protein is 0.012 C/m^2^. The translocation step of protein is 0.01 nm for all.

The pore currents for single, 2‐, and 3‐ protein translocations for both oblate and prolate situations are given in Figure 4. When the protein biosensor is more than 5 nm away from the pore, the calculated pore current equals the open pore current. The pore current decreases when the distance between the protein and the pore is less than 5 nm. For the single‐protein case, the pore current is minimal when the widest part of the protein passes through the pore. After that point, the current increases again as the protein progresses along the pore. For the 2‐protein model, the pore current continues to decrease between the widest parts of the first and second proteins and the lowest current value is observed at the broadest part of the second protein. The translocation time has increased. In contrast to the 2‐protein translocation, the pore current remains constant in the 3‐protein model after decreasing between the second and third proteins. As the number of proteins increases, the time to reach the open pore current after translocation also increases. Although the oblate and prolate results are similar, these results may vary for different protein models because there is not much difference between the a‐semiaxis and b‐semiaxis values of the protein model used. As the difference between these two values increases, the way the protein passes through can be estimated by examining the ion current. As the protein is affected differently in different locations, it diffuses at different speeds. For example, although it moves with the ions moving with the electric field outside the nanopipette, its speed changes due to the surface effect of the glass material after it enters the nanopipette. At this point, the number of ions that move together with it also decreases because it will block the pore. The ion current data points were calculated based on the location information in the simulation. Calculations were made independent of time. According to the literature, the protein moves faster during entry and exit and slows down during translocation (Perry, Momotenko, et al., 2016).

The protein modeling assumed a smooth ellipsoid shape with continuous and smooth surfaces. The simulations were performed with SCD of 0.012 and 0.065 C/m^2^ for fine‐tuning. The current drop value is closely related to the SCD of the protein, and as the SCD increases, the current drop also increases. Fractional blockades (the ratio of current difference during the translocation and open pore current), commonly used in protein discrimination, were calculated. This value is calculated using the ratio of the pre‐pore open pore current to the minimum current during pore formation and translocation. For single, 2‐, and 3‐protein translocations in the oblate model, the fractional blockades are 42.06%, 61.91%, and 75.59%, respectively, whereas for the prolate model, the values are 38.47%, 56.99%, and 66.31%, respectively. The fractional blockade increases for both oblate and prolate models, and the current drop is greater in the oblate model because the pore is more closed. The difference in fractional blockade values increases as the number of bound proteins increases. The difference in fractional blockade values is approximately 3% for single‐protein translocation, about 5% for 2‐protein translocation, and about 9% for 3‐protein translocation. These results suggest that the protein translocation shape is an important parameter.

**FIGURE 5:**
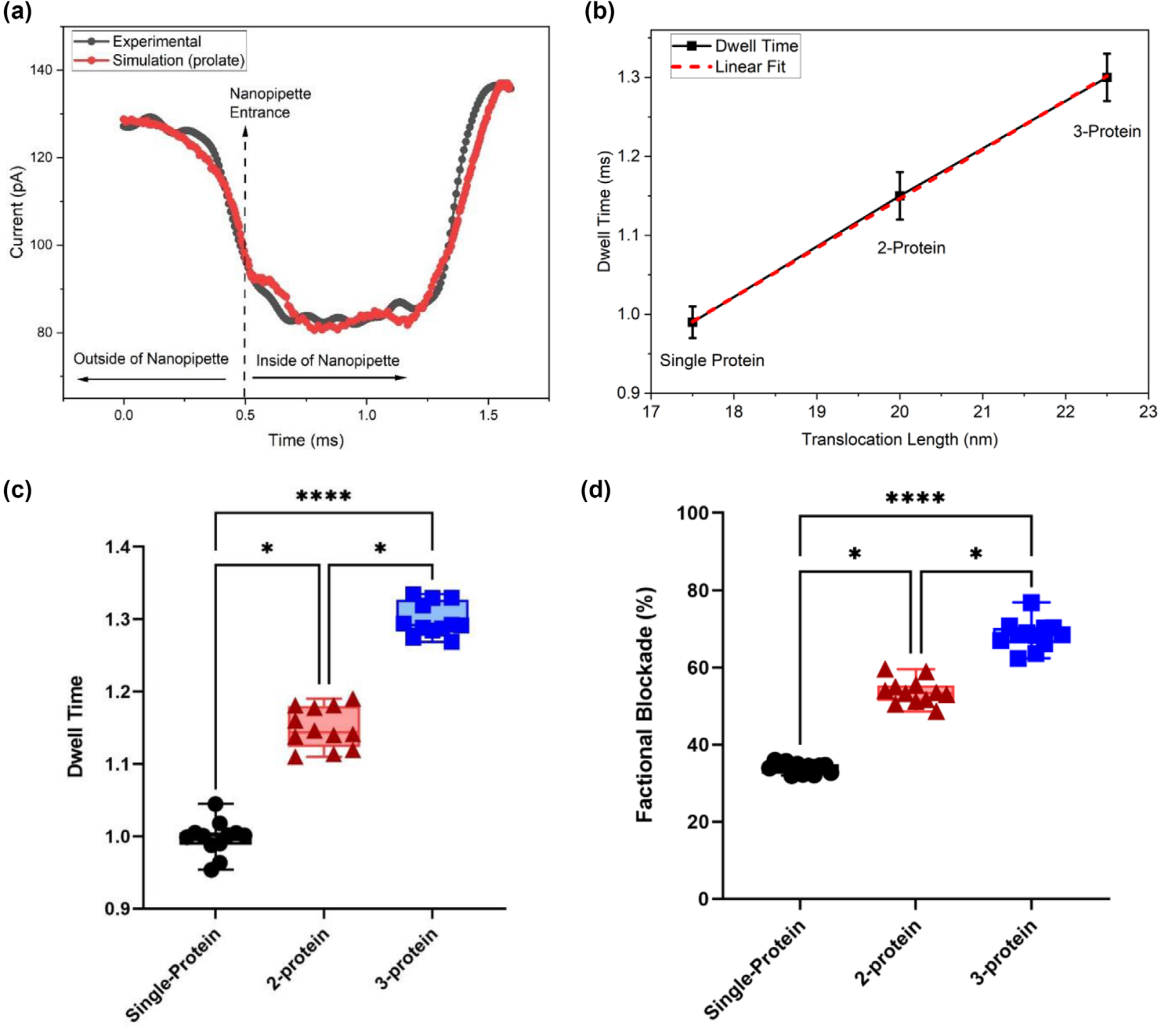
Comparing experimental and simulation results and conducting a comparative analysis of protein translocation models. In (a), a comparison between experimental and simulation results is presented. The protein's surface charge density (SCD) is determined to be 0.065 C/m^2^. Simulation results for the 3‐protein prolate model are highlighted, revealing that single‐protein and 2‐protein simulation results exhibit shorter dwell times. (b) Showcases the results of a protein translocation speed analysis with linear fitting applied. Notably, the highest speed is observed in single‐protein translocation. (c and d) illustrate the outcomes of statistical analyses for dwell time and fractional blockade, respectively. These analyses were conducted on a dataset comprising 12 protein translocations for each model, providing a comprehensive evaluation of the differences between these models. *p < .02 and ****p < .0001, by Kruskal–Wallis test with the original Dunn's method for multiple comparisons.

In Figure 5a, the graph compares experimental and simulated results. The minimum mean square error (MSE) value was found in the 3‐protein prolate model. This indicates that the predictions of this model closely match the actual values, with minimal deviation when compared to the single and 2‐protein models. The average speed of protein during translocation was calculated as 17.68, 17.39, and 17.31 μm/s for single, 2‐, and 3‐protein models, respectively. Dwell time (translocation time) was calculated based on when the current value began to change within ±0.5% of the measured current value before and after translocation. As the number of proteins moving together increases, the translocation speed decreases. Figure 5b shows the linear relationship between the average speed of single, 2‐, and 3‐protein translocation. This relationship suggests that the translocation process is rate‐limited by a single step, likely to be the movement of the translocating protein through the nanopore channel. This relationship can be used to calculate the translocation rate, which measures how quickly the protein moves through the channel. The linear relationship between translocation length and dwell time is also significant because it suggests continuous translocation. This contrasts with other translocation models, which propose that the process occurs in discrete steps. Figure 5c,d presents detailed visual representations of our statistical analyses for dwell time and fractional blockade, which offer profound insights into the behavior of protein translocation models. These exhaustive assessments were carried out meticulously on a substantial dataset comprising 12 protein translocations for each model. All statistical analyses were diligently conducted using GraphPad Prism 9 (GraphPad Software). A significance level of *p* < .05 was firmly established for determining statistical significance. We expertly utilized the ordinary Kruskal–Wallis test, a well‐suited approach for non‐normally distributed data. Following this, post hoc multiple comparisons were meticulously performed using the original Dunn's test further to elucidate the intricate statistical relationships between individual datasets.

The pore current during translocation is not constant and shows ±5.3% fluctuations for the 3‐protein model, whereas this value is ±2.8% for the single‐protein model. The fluctuation in pore current decreases as the number of proteins decreases. The fluctuation in pore current during translocation is related to the protein shape and amino acid sequence. This is because the protein must fit through the pore in order to be translocated, and the shape and sequence of the protein will determine how easily it can pass through the pore. The pore current is measured as the flow of ions through the pore. When a protein is translocating through the pore, it can block the flow of ions, which causes a decrease in the pore current. The magnitude of the decrease in pore current is related to the size and shape of the protein, as well as the charge distribution on the protein surface.

The protein's amino acid sequence also affects the pore current fluctuation during translocation. This is due to variations in the charge levels of different amino acids, and the distribution of these charges on the protein surface can impact the ease with which the protein traverses the pore. The fluctuation in pore current during translocation can be used to study the properties of proteins, such as their size, shape, and charge distribution. This information can be used to understand better how proteins function and to develop new drugs and therapies. The primary constraint in this perception is the SNR; for single‐protein amino acid sequencing, this ratio should be ≥3.

Based on dwell time and fractional blockade values for single, 2‐, and 3‐protein translocation in raw current data, discrimination can be made. The fractional blockade is 33%−36% for the single protein, 52%−57% for the 2‐protein model, and 61%−70% for the 3‐protein model. The discrimination process can be visualized in two dimensions using dwell time values, which are 1.0 ± 0.02 ms for the single protein, 1.15 ± 0.03 ms for the 2‐protein model, and 1.3 ± 0.03 ms for the 3‐protein model.

## 4 CONCLUSION

Moving proteins through tiny biosensors called nanopipettes is being studied using simulations to mimic the transport of one or more proteins for proteomics. The results of these simulations are then compared to experimental data. The simulation results showed that the distance between the protein and the pore affected the pore current, and the pore current decreased as the protein approached the pore. In the single‐protein translocation, the minimum pore current was observed when the widest part of the protein passed through the pore, whereas in the 2‐protein translocation, the lowest pore current was observed at the broadest part of the second protein. In contrast to the 2‐protein translocation, the pore current remained constant after decreasing between the second and third proteins in the 3‐protein model. The fractional blockade increased as the number of proteins increased, and the current drop was greater in the oblate model. The simulation results showed that the protein translocation shape was an important parameter. The minimum MSE value was obtained in the 3‐protein prolate model. The fluctuation in pore current during translocation can be related to the protein shape and amino acid sequence.

The results indicate that the fractional blockade and dwell time can separate single, 2‐, and 3‐protein translocation based on the current values measured experimentally. Thus, it allows us to distinguish multiple protein translocations on raw current data, which adversely affects the single‐protein sequencing studies, and to select only single‐protein blockades. The SNR should be above 3 for amino acid sequencing of a single protein. The simulation results also suggest that protein translocation through a nanopore biosensor is affected by several factors, such as the distance between the protein and the pore, the shape of the protein, and the number of proteins moving together. These findings may provide valuable insights for designing and optimizing nanopore biosensors for single‐protein analysis and sequencing. This has direct relevance in early disease diagnosis, enabling quicker and more reliable detection of biomarkers. The broader impact of our work is felt in developing enhanced biosensors with superior selectivity, sensitivity, and precision, facilitating breakthroughs in single‐cell analysis, and contributing to the evolution of cutting‐edge technologies with profound implications for both research and clinical applications.

## AUTHOR CONTRIBUTIONS

The author confirms sole responsibility for the following: study conception and design, data collection, analysis and interpretation of results, and manuscript preparation.

## CONFLICT OF INTEREST STATEMENT

The author declares no conflicts of interest.

## FUNDING INFORMATION

This research received no specific grant from any funding agency in the public, commercial, or not‐for‐profit sectors.

### PEER REVIEW

The peer review history for this article is available at https://publons.com/publon/10.1002/brb3.3405

## Data Availability

The datasets generated and analyzed during the current study are not publicly available but are available from the corresponding author on reasonable request.
